# Intestinal Barrier Function in Health and Disease—Any Role of SARS-CoV-2?

**DOI:** 10.3390/microorganisms8111744

**Published:** 2020-11-06

**Authors:** Lakshya Sharma, Antonio Riva

**Affiliations:** 1 Faculty of Life Sciences and Medicine, King’s College London, London SE1 1UL, UK; lakshya.sharma@kcl.ac.uk; 2 Foundation for Liver Research, Institute of Hepatology, London SE5 9NT, UK

**Keywords:** gastrointestinal, COVID-19, SARS-CoV-2, microbiota, gut barrier, gut permeability, gut–liver axis, immune, FMT, probiotics

## Abstract

Alterations in the structure and function of the intestinal barrier play a role in the pathogenesis of a multitude of diseases. During the recent and ongoing coronavirus disease (COVID-19) pandemic, it has become clear that the gastrointestinal system and the gut barrier may be affected by the severe acute respiratory syndrome coronavirus 2 (SARS-CoV-2) virus, and disruption of barrier functions or intestinal microbial dysbiosis may have an impact on the progression and severity of this new disease. In this review, we aim to provide an overview of current evidence on the involvement of gut alterations in human disease including COVID-19, with a prospective outlook on supportive therapeutic strategies that may be investigated to rescue intestinal barrier functions and possibly facilitate clinical improvement in these patients.

## 1. Introduction

Infection with SARS-CoV-2 is responsible for causing COVID-19, which has been classified as a pandemic by the World Health Organisation. Although SARS-CoV-2 has been shown to largely impact the respiratory system, SARS-CoV-2 is also able to interact with the gut. However, the relevance of these interactions to disease pathogenesis and severity is still unclear.

The gut barrier is essential in maintaining intestinal homeostasis and, when damaged, leads to the development of pathology. There has been association between gut barrier damage and many different disease states. As a result, therapies aimed at restoring a damaged gut barrier have been proposed.

## 2. Anatomy and Physiology

The gut is organised into distinct anatomical layers, each of which plays an important role in allowing the gut to function as a barrier to foreign material, such as pathogens and other noxious stimuli.

### 2.1. Mucus and Microbiome

Mucus is composed of a relatively thick layer of highly glycosylated mucin proteins, which are produced by the goblet cells present in the intestinal epithelium. The mucus layer thickness increases between the small intestine and the colon [1], and this is key for its protective functions along the intestinal tract. This mucus layer protects the epithelial cells from damage by bacteria and large molecules, such as food particles [2], by acting as a physical barrier. The mucus layer also contains immunoglobulins A (IgA) and autochthonous bacteria, both of which are beneficial in preventing the over-growth of pathogenic bacteria.

In particular, commensal bacteria are thought to have a crucial role in preserving the intestinal barrier. This is partly due to their ability to compete with, and thus limit gut colonisation by, pathogenic organisms [3]. Moreover, products of commensal bacteria fermentation are also involved in maintaining the gut barrier. Short-chain fatty acids (SCFAs), such as butyrate, are able to support intestinal barrier functions in many ways [4]. One proposed mechanism is that butyrate is able to regulate tight junctions [5], leading to a reduction in epithelial permeability.

Below the mucus layer, a second layer of highly glycosylated proteins, the glycocalyx, lines the surface of epithelial cells. Similarly to the mucus layer, these cell membrane-bound glycoproteins provide a physical barrier that prevents pathogenic microorganisms from interacting with the gut epithelial cellular monolayer [6].

### 2.2. Epithelium and Tight Junctions

**Figure 1:**
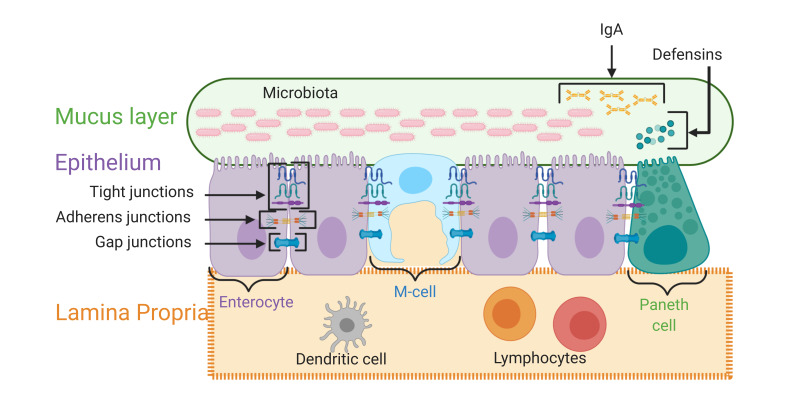
This figure outlines the key layers of the intestinal barrier. The mucus layer is composed of mucin proteins, but this layer also contains IgA antibodies, defensins and a proportion of the intestinal microbiota. The epithelial layer contains many specialized cell types, including enterocytes, microfold cells (M-cells) and Paneth cells, amoung others. The cells in this layer are connected by transmembrane protein complexes. The lamina propria is a connective tissue layer that contains immune cells, blood vessels and lymphatic vessels. Figure created with BioRender.com (accessed Aug 2020).

The most common cell type in the mucosal epithelial layer are enterocytes (or intestinal epithelial cells, IECs), forming 90% of cells in this monolayer [7]. Enterocytes are a site of absorption, but also serve as a key component of the gut barrier. Figure 1 provides a description of the key gut barrier layers.

Transmembrane protein complexes, such as tight junctions (TJs) and adherens junctions (AJs), enable the monolayer structure of the intestinal epithelium to be maintained; additional intercellular junctions called gap junctions further strengthen this structure. Tight junctions are the most apical type of intercellular junctions, are highly dynamic, and determine intestinal paracellular transport. Adherens junctions and, further, gap junctions are found more basolaterally.

Both TJs and AJs comprise transmembrane ‘adhesive’ proteins, which mediate actual intercellular contact, and cytosolic ‘proximal’ proteins, which anchor the junctional complex to the intracellular environment and the cell cytoskeleton [8,9,10]. For TJs, the main adhesive proteins are occludin and claudins, whilst the main proximal proteins are the ‘zonula occludens’ proteins (ZO-1, ZO-2, and ZO-3) [8,9,10,11]. For AJs, the main adhesive proteins are the cadherins, such as epithelial (E)-cadherin, whilst the main proximal proteins are the catenins (alpha, beta and delta) [8,9,10].

Tight junctions, as the main complexes mediating intercellular contact, are heavily regulated. Zonulin, for instance, is an established TJ modulator. It is thought that an increase in zonulin concentration can cause ‘zonula occludens’ proteins to disengage [12] and thus disrupt the TJ complex. This change is thought to lead to increases in paracellular permeability. A large variety of other TJ regulators have also been identified [13], including pro-inflammatory cytokines such as tumour necrosis factors (TNF) and interleukin-13 (IL-13). These cytokines are thought to be able to regulate TJ function by acting on myosin light chain kinase (MLCK). While other mechanisms have been suggested, it is widely proposed that activation of MLCK is a common final pathway in TJ regulation, especially in the acute phase [14].

### 2.3. Mucosa-Associated Immune System

Both epithelial cells and a variety of gut-resident immune cells provide local protection against translocated pathogens or metabolites.

Specialised epithelial cells provide a noteworthy contribution towards protecting the epithelial layer. Paneth cells, a type of epithelial cell, produce defensins and other antimicrobial peptides. Defensins can be found in the mucus layer and assist in defending the gut against pathogens. Paneth cells, along with other cells of the epithelial layer, are thought to form a key part of the gut barrier [15].

Peyer’s patches form a part of the gut-associated lymphoid tissue (GALT) and contain microfold cells (M-cells). M-cells are one of the cells that are important for antigen sampling [16], which can initiate a pathway that ultimately leads to the activation of T and B lymphocytes. Once activated, plasma cells in the lamina propria can secrete IgAs, which are then actively transported into the gut lumen [17] and can be found in the mucus layer.

While components of the gut immune system can be found in concentrated distinct structures, such as Peyer’s patches, other types of resident immune cells can also be found disseminated along the lamina propria. These cells include, for example, mucosal-associated invariant T cells (MAIT), which are directly antibacterial and can also participate in bystander immune regulation [18,19,20,21]; regulatory T cells (Tregs), which participate in limiting indiscriminate immune and inflammatory activation and play a role in maintaining immune tolerance to commensal and food antigens [20,22,23]; and innate lymphoid cells (ILC), which shadow T cells with their own innate-like functions and collaborate actively in shaping the local intestinal immunity [20,24,25,26,27].

## 3. Mechanisms of Pathology

**Figure 2:**
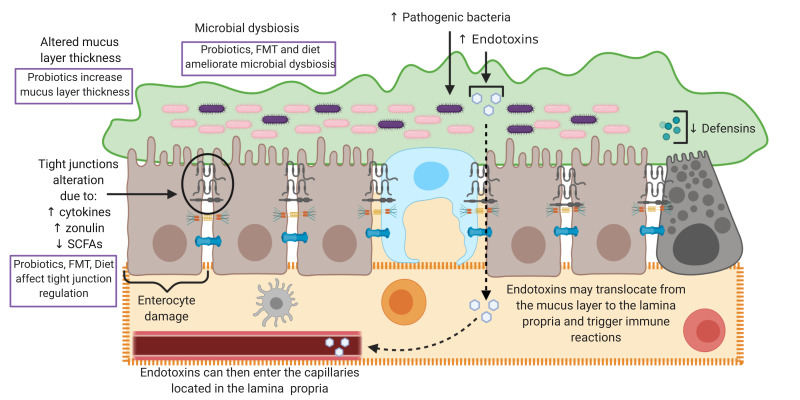
This figure illustrates how pathology can arise in the different layers of the gut barrier and highlights how examples of treatment strategies can ameliorate these pathological alterations. In pathology, there can be an alterations of mucus layer thickness. Probiotics have been shown to increase mucus layer thickness. Microbial dysbiosis can occur in disease and it is thought that probiotics, faecal microbiota transplantation (FMT) and dietary therapies can correct this dysbiosis. Tight junction alterations can also occur in disease and this has been associated with an increase in cytokines, an increase in zonulin and a decrease in short-chain fatty acids (SCFAs). Probiotics, FMT and dietary therapies could potentially reverse these changes by increasing the concentrations of SCFAs. In the figure, small arrows indicate increase or decrease, while the dotted arrows indicate the movement of bacterial matter as it translocates across the intestinal barrier. Figure created with BioRender.com (accessed on August 2020).

Pathology can arise in any of the gut barrier layers. Alteration in these layers can cause increased intestinal permeability. Figure 2 summarises mechanisms responsible for gut barrier alterations and indicates areas where therapeutic strategies may ameliorate barrier dysfunctions.

### 3.1. Mucus and Microbiome

Alterations in the secretion of mucin by goblet cells can lead to an abnormal intestinal mucus layer. Certain pathogens are able to disrupt mucin secretion and impact the consistency of the mucus layer [28]. For example, certain bacteria and viruses can lead to the secretion of mucin-degrading enzymes [29]. This can limit the functionality of mucus as a barrier and thus increases susceptibility to increased gut permeability.

Additionally, decreases in mucin hydrophobicity can be a marker of barrier dysfunction [30]. It has been suggested that this alteration in the mucus layer can be associated with gut ischemia [31]. This again suggests that alterations in the mucus layer can impact the permeability of the gut barrier.

Commensal bacteria are increasingly being considered as an integral aspect of the gut barrier [7], and the mucus layer and microbiome are closely associated. Therefore, alterations in one can lead to pathology in the other [32]. A change in the composition of the microbiome (dysbiosis), for instance, can alter the production of SCFAs such as butyrate. This can result in impaired TJ regulation, and thus lead to increased intestinal permeability [4]. For example, a well-known cause of intestinal microbial dysbiosis, chronic alcohol consumption, has been shown to be correlated with elevated serum endotoxin and other surrogate markers of bacterial translocation [18,33,34], demonstrating a link between microbial dysbiosis following alcohol consumption and increased permeability. Additionally, alcohol consumption can also affect the mucus layer itself by causing a reduction in its thickness [35].

### 3.2. Epithelium and Tight Junctions

Mucosal epithelial cells form an integral part of the gut barrier and are predominantly affected in gut barrier pathology, as are the TJs in-between these cells. Functional TJs are necessary in maintaining an effective level of paracellular and transcellular permeability [14]. Thus, it is thought that some pathophysiological mechanisms that can impact TJ regulation can cause an increase in intestinal permeability [36]. Some examples of factors that are thought to employ mechanisms which can cause increased permeability by affecting TJs and the epithelial cell layer include pathogenic organisms, alcohol and non-steroidal anti-inflammatory drugs (NSAIDs).

#### 3.2.1. Pathogenic Organisms

Pathogenic bacteria can interfere with TJs and cause increased intestinal permeability. As mentioned previously, intestinal dysbiosis can lead to altered SCFA/butyrate metabolism and impaired TJ regulation. In addition to this, pathogens can interact with epithelial TJs directly, through a variety of proposed mechanisms [37], to cause intestinal barrier dysfunction. For example, secretions of ‘zonula occludens’ toxin by *Vibrio cholerae* has been associated with changes in TJ occludins and subsequent increase in epithelial permeability [38].

Furthermore, epithelial cells can undergo apoptosis following infection by pathogenic bacterial species. The mechanism by which this occurs is thought to be linked to an increase in TNF in response to bacterial infection [39]. This epithelial damage would result in a transcellular permeability increase, and it has indeed been demonstrated that translocation of epithelial cell and junctional markers into the systemic circulation increase upon intestinal damage, such as fatty acid binding protein 2 (FABP2) [40,41,42] or claudin-3 [42,43,44].

As previously mentioned, bacteria (pathogenic or commensal) and their metabolites can enter the systemic circulation during times of increased intestinal permeability [45]. An association has been demonstrated between elevated physiological stress states and increased serum endotoxin levels [46]. However, it is important to note that the exact molecular mechanisms by which translocations of bacteria and endotoxins occur is still unclear and partly controversial [47,48].

As well as pathogenic bacteria, viruses may also play a role in disrupting the intestinal epithelium. For example, amongst coronaviruses, Middle East respiratory syndrome-related coronavirus (MERS-CoV) has been shown to disrupt the function of intestinal epithelial cells in animal models [49], which could increase the risk of increased intestinal permeability due to enterocyte damage. Additionally, it has been shown that SARS coronavirus (SARS-CoV) can impact PALS1, a TJ protein found in the intestinal and lung epithelium [50]. Recently, it has been proposed that SARS-CoV-2 may increase intestinal permeability, potentially by causing damage to enterocytes and the epithelial layer [51]. While this does suggest that coronaviruses can impact TJs, definitive evidence is—however—still limited and warrants further molecular research.

#### 3.2.2. Alcohol

A correlation has been identified between alcohol consumption and increased epithelial damage [52]. Alcohol metabolites, such as acetaldehyde, can have damaging effects on epithelial cells directly, via reactive oxygen species [52], but they may also alter the expression of TJ proteins [53]. The enteropathy that arises due to alcohol can result in increased transcellular permeability.

#### 3.2.3. NSAIDs

The mechanism by which NSAIDs are able to cause epithelial layer damage is still not fully established, but as with pathogenic bacteria and alcohol, it is likely to be multifaceted. Studies have suggested that NSAIDs may inhibit autophagy in intestinal epithelial cells [54], which would prevent the recycling of cellular organelles and ultimately result in enteropathy.

Furthermore, the impact of NSAIDs on epithelial TJs has been investigated as another possible mechanism leading the NSAID-mediated increased permeability [55], but no concrete molecular mechanism of action has been conclusively confirmed.

### 3.3. Immune Activation and Alterations

A wide variety of external and internal stimuli can interact with the mucosal immune system and cause an increase in gut permeability; vice versa, alterations in gut permeability and increased bacterial translocation can cause mucosal and systemic immune dysfunctions.

A well-known agent that can influence the mucosal immune system is alcohol. In addition to other mechanisms, it is thought that alcohol can increase intestinal permeability by suppressing the production of defensins by Paneth cells [52]. This can decrease the ability of the intestinal barrier to combat pathogenic bacteria, thus facilitating the production of excess endotoxins. These may then have the potential to translocate and enter the lamina propria, where they may trigger an immune response. As a part of this response, inflammatory cytokines can be released into the circulation and exert systemic effects [56,57]. In fact, during alcohol-related cirrhosis and alcoholic hepatitis, conditions known to be associated with increased gut permeability, markers of bacterial translocation and pro-inflammatory cytokines are increased in the systemic circulation [18,34,58]. This is also seen in acutely decompensated cirrhosis, where these markers appear to be raised both systemically and also locally in the intestinal lumen, strengthening the links between local intestinal alterations and systemic immune imbalance [57]. Furthermore, it has been previously demonstrated that during alcohol-related liver disease, bacterial translocation across a damaged gut barrier can strongly affect systemic immunity (i) by promoting upregulation of inhibitory immune checkpoint receptors (PD-1 and TIM3) and secretion of anti-inflammatory IL-10 from circulating T cells [34]; (ii) by suppressing T-cell production of antibacterial IFN-g [34]; and (iii) by directly inhibiting antibacterial functions in neutrophils and MAIT cells [18,34].

Besides alcohol and bacteria, viruses are also responsible for local immune alterations in the gut. For instance, infections with the human immunodeficiency virus (HIV) have been associated with gut barrier damage [59,60]. A possible reason for this is that patients infected with HIV may not be able to repair a damaged gut barrier as effectively, due to a depletion of Th17 cells in their GALT [61].

## 4. Associations with Disease

Disruption of the intestinal barrier and increased intestinal permeability have been associated with a variety of diseases. A vast range of diseases and disease mechanisms have been linked to intestinal permeability changes. However, it should be noted that there has been difficulty in conclusively establishing a causal relationship between increased intestinal permeability and disease [2].

### 4.1. Intestinal Disease

Alterations in the mucus layer are thought to play a role in increased colonic permeability during ulcerative colitis (UC). As goblet cells are responsible for the production of mucin, it would be plausible that goblet cell changes contribute towards altered mucin expression and it has been shown that patients with UC and Crohn’s disease have significantly decreased Goblet cell proportions [62]. Patients with active UC have been found to have qualitative modifications in a type of mucin, MUC2, including a decrease in complex glycans and an increase in smaller glycans [63]. There have also been indications of alterations in the quantity of mucin production in both UC and Crohn’s disease patients [64]. As a result of these changes in the mucus layer, it has been proposed that defensins will not be fixed to the mucus as effectively [65], which can lead to a weakened responses against pathogens and an overall disturbance in gut barrier function.

Additionally, a decrease in Paneth cell function has also been linked to the pathogenesis of Crohn’s disease [66]. As Paneth cells are responsible for the production of defensins, a decrease in Paneth cell concentration may lead to a subsequent decrease in defensin production. This could result in a weakened gut barrier that is less equipped to combat pathogens.

The increase in inflammatory cytokines, such as TNF-α, that occurs in Crohn’s disease may impact the integrity of epithelial cells and TJs. An increase in TNF-α is thought to induce enterocyte apoptosis and lead to inappropriate cell-shedding [67], which has the potential to increase transcellular permeability. In vitro studies have shown that TNF-α increases may also be associated with alterations in the regulation of the TJ proteins, for example claudin-2 in Crohn’s disease [68,69]. Ultimately, the ‘exact’ mechanism by which Crohn’s disease is associated with an increase in intestinal permeability is unknown, but it is thought that mucus layer changes, Paneth cell depletion, epithelial damage and TJ alterations play a role in the pathogenesis of the disease.

Patients with active coeliac disease are also thought to have alterations in their expression of TJ proteins. An increase in intestinal permeability after gluten exposure, in coeliac disease patients, has been demonstrated through ex-vivo human studies [70]. It is thought that this change in permeability may be a result of an increase in zonulin release, following exposure to gliadins [71], which are components of gluten. As previously mentioned, zonulin is an established TJ modulator [72], and its increased levels in patients with active coeliac disease may facilitate increased paracellular permeability. Moreover, associations between variants in the TJ genes, PARD3 and MAGI2, and coeliac disease have been revealed [73]. This further supports the idea that TJ alterations play a role in the pathogenesis of coeliac disease.

### 4.2. Gut–Liver Axis and Biliary Conditions

Increased intestinal permeability is thought to have an association with liver disease, as a result of gut–liver axis disruption [74]. Patients with non-alcoholic fatty liver disease (NAFLD) have been shown to have significantly increased intestinal permeability, in comparison to healthy controls, which has been associated with changes in expression of the TJ proximal protein zonula occludens-1 (ZO-1) [75]. This increase in intestinal permeability is likely to be even more prevalent in those patients who have developed non-alcoholic steatohepatitis (NASH) [76]. In fact, serum markers of intestinal permeability, bacterial translocation and subsequent immune activation are detectable systemically in these patients [77,78,79,80,81,82,83]. Additionally, patients with liver cirrhosis have been shown to have altered TJ protein expression, with one study finding a significantly lower expression of occludin and claudin-1 in particular [84]. The modifications of TJ protein expression seen in chronic liver disease could result in increased paracellular gut permeability. During decompensated cirrhosis, compared to stable disease, markers of bacterial translocation, epithelial cell damage and immune activation are increased and detectable in both the systemic circulation and in the intestinal lumen [57]. During alcohol-related liver disease (ALD), increased intestinal permeability and bacterial translocation are hallmarks of disease progression [58], and we and others have demonstrated increased systemic intestinal markers in multiple cohorts of ALD patients [18,34].

Furthermore, abnormal composition of the gut microbiota is a key feature of NAFLD, liver cirrhosis and alcoholic liver disease (ALD) [35,85]. Increases in secondary bile acids and pathogen-associated molecular patterns (PAMPs), such as endotoxin, and decreases in SCFAs have been proposed as mechanisms through which this altered bacterial composition is thought to lead to defects in the intestinal barrier and contribute to the pathogenesis of those liver conditions [86].

Gut barrier alterations have also been discussed with regards to chronic cholestatic liver diseases, primary biliary cholangitis (PBC) and primary sclerosing cholangitis (PSC) [87,88]. Increases in intestinal permeability and the presence of significant endotoxemia has been found in association with PBC [89], and although one study found that patients with PSC did not experience significant increases in intestinal permeability [90], there is a clear association between PSC and intestinal disease [91], so conducting further research in this area may be helpful in improving understanding about the impact of intestinal permeability on PSC.

### 4.3. Metabolic Disease

Metabolic conditions, such as obesity and diabetes mellitus, have been linked to increases in intestinal permeability [92,93,94,95]. It has been found that weight reduction therapy can reduce the intestinal permeability of obese individuals to normal levels [96], suggesting that there may be an association between obesity and intestinal permeability. It has been postulated that microbial dysbiosis could lead to increases in intestinal permeability by impacting TJ expression in obese patients [97].

Similarly, there is evidence suggesting a correlation between type 2 diabetes mellitus (T2DM) and increased intestinal permeability [98,99]. In addition to this, it has been suggested that patients with T2DM who have poor glycaemic control are at a greater risk of having a damaged intestinal barrier [100]. As with obesity, microbial dysbiosis is also thought to influence the pathogenesis of increased permeability in T2DM, as a result of reductions in SCFAs [37]. Type 1 diabetes mellitus (T1DM) patients are also thought to experience increased intestinal permeability due to unclear mechanisms, that could potentially involve alterations in zonulin concentrations and in the microbiota [7].

### 4.4. Gut–Brain Axis

There is constant interaction between the brain and the gut microbiome. It is thought that the factors altering intestinal permeability may impact the permeability of the blood-brain barrier in a similar way [101]. For example, in their review, Camara-Lemarroy highlight that alterations in the gut microbiome may lead to increased intestinal permeability and decreased SCFA production, which can then impact permeability of the blood-brain barrier and cause neuronal immune-related dysfunction [102]. The authors were particularly interested in the association between intestinal permeability and multiple sclerosis. They suggested that further research in this area would help gain insight into the multifaceted pathogenesis pathways of multiple sclerosis. The call for further research into the relationship between intestinal permeability and neurological conditions was echoed by Obrenovich, who highlighted shortcomings in the currently available studies regarding this topic [103].

### 4.5. Functional Disorders of the Gut

The notion of ‘leaky gut syndrome’ has been widely debated. The general premise of ‘leaky gut syndrome’, that a damaged gut barrier can lead to an increase in PAMPs, such as endotoxins, entering the circulation, is likely to be accurate, but it has been suggested that it is improbable for this to occur through a paracellular route of permeability [48]. Furthermore, advocates of ‘leaky gut syndrome’ have identified it as the cause of a variety of different diseases. Many of the diseases associated with ‘leaky gut syndrome’ are functional disorders or have complex and unclear pathogenesis pathways, such as food intolerance, fibromyalgia, chronic fatigue syndrome and autism [104]. It should be noted that there is limited data to suggest causal relationships between ‘leaky gut syndrome’ and these conditions. Additionally, there have been claims that resolving ‘leaky gut syndrome’ can cure the diseases it has been associated with. There is currently limited conclusive evidence to show that decreasing intestinal permeability has an effect on disease progression [105]. However, treatments aimed at decreasing intestinal permeability are being increasingly explored, with particular attention being paid to the effect these treatments have on disease symptoms.

## 5. Treatments

Considering the wide range of diseases that have associations with a damaged gut barrier, efforts have been made to explore potential treatments to restore gut barrier function. Many of these treatments look to reduce increased intestinal permeability (Figure 2).

### 5.1. Faecal Microbiota Transplantation

Faecal microbiota transplantation (FMT) is a therapy that involves the transfer of faecal bacteria from one individual to another. The association between FMT and intestinal barrier function has been explored. Multiple animal studies have found that treatment with FMT correlates with a decrease in intestinal permeability [106,107].

FMT is currently primarily used to treat diarrhoea symptoms associated with *Clostridioides difficile* infections or other conditions, such as ulcerative colitis. However, FMT is also being used in liver conditions, with results showing symptom improvement following treatment [92]. A randomised-control trial conducted in patients with non-alcoholic fatty liver disease (NAFLD), demonstrated that treatment with FMT is able to significantly decrease intestinal permeability in NAFLD patients with elevated permeability levels [108]. This is one of the first studies that has been able to demonstrate, in humans, that FMT treatment can lead to a significant reduction in intestinal permeability.

The mechanism by which FMT is able to decrease gut barrier permeability is not clear. One possible mechanism can be linked to the increase in short-chain fatty acids (SCFAs) following FMT treatment, in particular butyrate, as demonstrated by Dutta et al. in patients with recurrent *Clostridioides difficile* infections, thus regulating intestinal permeability by altering the expression of TJ proteins. This being said, an animal study that evaluated the effect of FMT on the TJ protein zonula occludens-1 (ZO-1) found that its expression was not altered following the intervention [109]. Therefore, more research is needed to confirm the association between FMT-related decrease in intestinal permeability and direct TJ regulation. It has also been suggested that FMT impacts the intestinal immune system by inducing the production of immunoglobulins [110]. Thus, the mechanism by which FMT is able to decrease intestinal permeability may also involve the gut immune system.

Other studies have proposed that using adjunctive probiotic treatment with FMT may lead to additional beneficial effects on the intestinal barrier, by conferring a stronger imprint to the production of microbial SCFAs [111].

### 5.2. Probiotics and Prebiotics

According to the definition by the World Health Organisation (WHO) and the Food and Agriculture Organisation of the United Nations (FAO), probiotics are “live microorganisms that, when administered in adequate amounts, confer a health benefit on the host” [112,113,114,115]. The impact of probiotic treatment on the gut barrier has been thoroughly investigated, but not consistently confirmed. While many studies have shown that probiotics can decrease intestinal permeability [116,117,118], others have found no significant change in permeability following probiotic treatment [119,120,121,122]. However, it should be highlighted that, to our knowledge, no studies have demonstrated a significant increase in intestinal permeability following treatment with probiotics.

More research is needed to conclusively confirm whether probiotics are able to strengthen the gut barrier and thus significantly decrease intestinal permeability, and to further understand the mechanism by which this effect could potentially occur. Various studies have tried to investigate this in depth. Firstly, it is thought that probiotics can improve intestinal barrier function by modifying the mucus layer. The probiotic *Akkermansia muciniphila* (*A. muciniphila*) is thought to lower permeability by increasing mucus layer thickness [123]. Secondly, probiotics are thought to affect TJ regulation. A study found that the serum concentration of the TJ regulator zonulin and intestinal permeability are both decreased following treatment with a probiotic [116]. Additionally, confocal laser scanning microscopy has shown that probiotics can stabilise the expression of the junction proteins occludin, claudin-1 and JAM-1 [118]. Moreover, microarrays have demonstrated that certain strains of the probiotic *Lactobacillus plantarum (L. plantarum)*, such as TIFN101, can regulate pathways involved in the transcription of genes that are related to TJ function [124]. Overall, various studies have demonstrated a change in TJ regulation following probiotic treatment, thus showing that probiotics could have a direct impact on the regulation of the gut barrier and intestinal permeability.

Unlike probiotics, prebiotics are not liver organisms but are defined as compounds or substrates that are “selectively utilized by host microorganisms conferring a health benefit” to the host (for example, fructans, galactans, inulin and some types of fibers) [115,125], either by supporting the growth of beneficial bacterial or by promoting their production of beneficial metabolites. It should be noted that there have been various studies that assess the effect of prebiotics on the gut barrier; however, many have not been able to demonstrate a significant change in intestinal permeability after treatment [126,127]. Following on from this, a study by Krumbeck et al. evaluated the effect that combined treatment with probiotics and prebiotics would have on intestinal permeability. They found that both treatments individually had the effect of decreasing intestinal permeability in obese patients receiving high-dose aspirin, but there was no significant additional decrease when a symbiotic was used [128].

Table 1 provides examples of randomised-controlled trials on the effect of FMT, probiotics and prebiotics on the intestinal barrier.

### 5.3. Dietary Treatment

There is interest in examining the role played by diet and lifestyle factors on intestinal permeability. Table 2 provides examples of randomised-controlled trials on the effect of dietary interventions on the intestinal barrier that have been published in the last ten years. Even though no definite causal link between dietary treatments and intestinal permeability has been established, there have been proposals regarding the pathways by which dietary fiber and peptides can strengthen the gut barrier.

The gut microbiome can increase production of SCFAs through fermentation of microbiota-accessible carbohydrates found in dietary fiber [134]. As discussed previously, increases in SCFAs have been associated with TJ regulation and thus can work to increase epithelial barrier function by lowering paracellular ‘leakiness’. In practice, studies that have assessed the effect of dietary fiber on intestinal permeability have found varying results [129,130].

Certain peptides and amino acids have also been associated with changes in intestinal permeability. It has been proposed that the casein peptide can regulate TJs by increasing the expression of occludin [135], which is thought to lead to a decrease in intestinal permeability. Additionally, amino acids, such as glutamine, have been found to lead to a decrease in intestinal permeability by some studies [131,132,136], possibly by affecting the regulation of TJ proteins directly and by preventing TJ injury from the alcohol metabolite acetaldehyde [137]. Glutamine is also thought to prevent disruption of the mucosal immune system by influencing IgA production, potentially through acting on the gut-associated lymphoid tissue [138]. It is important to note that, even though the aforementioned studies have found that glutamine can decrease intestinal permeability, there is no official recommendation for glutamine to be used as a supportive treatment option for hyper-permeability.

Dietary supplements have also been shown to improve gut barrier function and some have been proposed as potential treatments for hyper-permeability. Lamprecht et al. have tested the impact of zeolite supplementation on the intestinal barrier and have found a corresponding decrease in zonulin concentration [133]. As zonulin is a major regulator of TJs, a decrease in zonulin concentration is likely to be associated with a decrease in paracellular intestinal permeability. A change in measurements of intestinal permeability was also seen in supplementation with zinc [139], suggesting an improvement in intestinal barrier function; however, the validity of this study and its conclusions have been contested [140].

This being said, it has been difficult to establish significant causal relationships between diet and gut barrier function. One of the main reasons for this is that studies assessing dietary treatments are often done in outpatient settings, in which it can be difficult to regulate different control variables. Therefore, it may be worth considering dietary therapies as adjunctive and placing focus on other areas as primary therapies for increased intestinal permeability.

## 6. SARS-CoV-2 and the Gut

**Figure 3:**
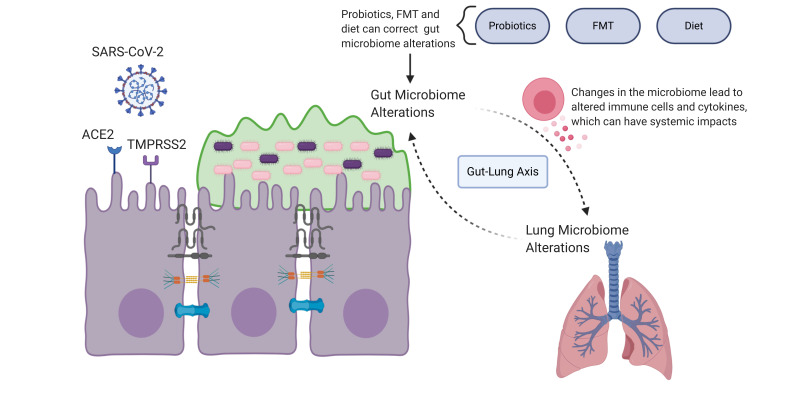
This figure delineates the link between SARS-CoV-2-related intestinal pathology and pathology associated with disruption of the gut–lung axis. Supportive treatment strategies to correct gut barrier alterations could potentially have a beneficial effect on rebalancing the gut–lung axis in COVID-19 patients. Figure created with BioRender.com.

Intestinal permeability has been explored in the literature for decades [141], and gut pathologies are a very current issue. In fact, SARS-CoV-2 has been shown to interact with the gut. Considering the relationship between SARS-CoV-2 and the gut can be beneficial in understanding the impact that gut barrier associated treatments could have on COVID-19 patients. Figure 3 delineates the link between SARS-CoV-2, gut alterations and the gut–lung axis, indicating possible areas where supportive therapeutic interventions may result as beneficial for COVID-19 patients.

### 6.1. Infection of Intestinal Cells

It has been shown that SARS-CoV-2 can infect intestinal organoids [142]. The virus utilizes the angiotensin I converting enzyme 2 (ACE2) receptor to gain entry into to cells [143] and there is a high level of ACE2 expression in enterocytes [144,145], suggesting that SARS-CoV-2 can take advantage of this and infect intestinal enterocytes. Furthermore, it is thought that transmembrane serine protease 2 (TMPRSS2), which is also highly expressed in enterocytes in the ileum and colon [145,146], is able to prime the spike protein of SARS-CoV-2 and facilitate the entry of the virus into cells [143].

Since ACE2 and TMPRSS2 are thought to enable SARS-CoV-2 to infect cells, interest has been shown in therapies that target the ACE2 and TMPRSS2 proteins, such as ACE2 fusion proteins and TMPRSS2 inhibitors [147], but there is currently limited available data regarding the efficacy of these treatments [148].

### 6.2. Gastrointestinal Symptoms and Clinical Considerations

Studies that have assessed the prevalence of gastrointestinal (GI) symptoms in patients infected with the SARS-CoV-2 virus have reported a wide range of results [149], although there seems to be a degree of consensus on the fact that a large proportion of GI symptoms are mild [150].

There is contrasting data on the relationship between GI symptoms and the disease progression of COVID-19. Some studies found that there is an increased risk of hospitalisation or clinical decline in patients with GI symptoms such as diarrhoea and nausea and/or vomiting [151]. In contrast, others have concluded that the severity of GI symptoms does not correlate with the severity of the clinical course of the disease [152,153,154,155]. In fact, one study found that the presence of GI symptoms was associated with a slower and less severe clinical disease progression [156]. Furthermore, some authors have suggested that people with pre-existing intestinal disease, such as inflammatory bowel disease (IBD), may not necessarily be at an increased risk of developing infections [157,158]. This being said, IBD severity can vary and not all patients with IBD will have the same level of risk of developing severe COVID-19 [159].

Even though it is not possible to say with certainty that GI symptoms affect disease course, they should still be monitored closely in patients. It has been found that GI symptoms may be the primary presentation for some patients infected with SARS-CoV-2 [160,161]. Thus, there is value in considering GI symptoms in relation to SARS-CoV-2 infection so that atypical presentations are not missed. Additionally, reports of persistent GI symptoms resulting in re-admission, following completion of treatment for pneumonia, have been described [162], which further emphasises that GI symptoms should not be ignored.

There has been increasing concern that SARS-CoV-2 may have a faecal–oral route of transmission, due to intestinal viral RNA shedding. Viral RNA has been found in stool samples of patients infected with SARS-CoV-2 [163]. Some studies have found that viral RNA in stool is more likely to be detectable if GI symptoms are present [164,165]. However, others have found no statistically significant link between viral RNA and increased severity of GI symptoms [163,166]. This being said, there has been suggestion that stool viral RNA can be positive even if the virus is not detectable through respiratory samples [164,167], which has led to the proposal that there may be value in considering stool sampling as a method of evaluating infection with the SARS-CoV-2 virus. Overall, despite some suggestion that the stool is unlikely to contain infectious viruses [168], it appears that further research is needed to confirm the risk of infection by faecal–oral transmission and its degree of importance in the context of overall transmission [169].

### 6.3. Microbiome

The gut microbiome of patients with COVID-19 has been shown to have considerably lower bacterial diversity and higher relative abundance of opportunistic pathogens compared to healthy controls, including *Streptococcus* spp., *Rothia* spp., *Veillonella* spp., and *Actinomyces* spp. [170]. The authors of this study proposed that these microbiome changes could be used as a diagnostic biomarker, but also highlighted its value as a target for treatment. Interest has been shown in therapies that impact the microbiome, such as faecal microbiota transplantation, probiotics and dietary treatments, for use as adjunctive therapies in the management of COVID-19 patients [171,172], as we will discuss subsequently.

One study identified a correlation between faecal microbiome alterations and increased COVID-19 disease severity. However, the causal nature of this relationship is not clear, in part due to confounding factors that are known to affect the gut microbiome, such as the exposure of COVID-19 patients to antibiotic therapy as they are often treated for suspected superimposed bacterial pneumonia. It has been suggested that the gut microbiome is important in the immune response to viruses and that microbial dysbiosis can lead to an inadequate immune response [173]. Additionally, animal models have shown that the microbiome can affect colonic ACE2 expression [174]. This would indicate that the composition of the microbiome can impact the ability of SARS-CoV-2 to infect enterocytes. Therefore, microbial dysbiosis of the gut could potentially predispose an individual to develop a more severe case of COVID-19. There is currently a pre-print study that supports this [175].

In addition to gut microbiome alterations, it has been shown that the microbiome of the airway is also disrupted in patients with COVID-19 [176]. This could potentially be related to the gut–lung axis, as we will discuss next.

Table 3 gives examples of studies that investigate the link between SARS-CoV-2 and gastrointestinal symptoms observed in COVID-19 patients (including alterations in the microbiome).

### 6.4. The Gut–Lung Axis

The gut—as discussed above—and the lung both have barrier layers that prevent pathogenic infiltration. The gut and lung microbiomes are thought to play an important role in maintaining this barrier by regulating the immune response [178].

A reduction in microbiome diversity has been associated with chronic respiratory diseases. For example, in cystic fibrosis patients have been shown to have alterations in both their lung microbiome and intestinal microbiome [179]. Lymphocyte migration is a potential mechanism by which the gut microbiome and lung microbiome can interact, which could impact systemic immunity and inflammation [178]. There has been suggestion that increased intestinal permeability may play a role in the pathogenesis of systemic inflammatory injury in COVID-19 [180].

Additionally, the SCFAs produced by commensal bacteria in the gut can potentially impact systemic immunity. It is thought that SCFAs can stimulate an anti-inflammatory or pro-inflammatory response, depending on the G protein-coupled receptor the SCFAs interact with [181]. In relation to this, it has been proposed that the microbiome is essential in maintaining immune homeostasis, which may be important to consider in patients with COVID-19 [182].

The inflammatory response to SARS-CoV-2 infection could be a factor determining variations in disease progression and GI symptoms. Although early studies did not find significant inflammatory changes following SARS-CoV-2 infection [154], subsequent studies observed increases in markers of inflammation, such as faecal calprotectin and IL-6, in patients infected by SARS-CoV-2 [177]. Furthermore, recent investigations focussed specifically on the intestinal inflammatory milieu in COVID-19 patients have demonstrated that intestinal infection with SARS-CoV-2 does indeed alter gut-specific inflammatory responses, interfering with local antiviral immunity and at the same time promoting increased secretion of pro-inflammatory cytokines; besides being detectable in faecal samples from some of these patients, this enhanced inflammatory response may correlate with immune regulation systemically [183,184,185]. In light of this, it will be valuable to investigate in more detail the link between the gut microbiome, the intestinal infection with SARS-CoV-2 and the modulation of local and systemic inflammation, due to the important role that this is thought to play in COVID-19 disease pathogenesis and outcome [186].

### 6.5. Gut Barrier Treatments and SARS-CoV-2

Many of the treatments that have been used to treat a damaged gut barrier have also been proposed as adjunctive therapies in SARS-CoV-2 infection.

For example, based on the observed interaction between SARS-CoV-2 and the intestine, with development of GI symptoms and microbial dysbiosis, it has been suggested that FMT could be explored as a therapeutic tool for patients with COVID-19 [171].

Furthermore, there has been some evidence to show that certain probiotics can be used to control viral infections [180]. This has led some to suggest that probiotics could be considered as an adjunctive therapy in the treatment of COVID-19 patients [172]. In fact, one study found that, in patient with SARS-CoV-2 infection, adjunctive treatment with probiotics was associated with a significant decrease in the risk of developing respiratory failure [187]. This improvement in clinical outcome could be due to the antiviral and anti-inflammatory effects that probiotics are thought to have [188,189]. In addition, probiotics are often regarded as being safe, even for vulnerable patients, and this—together with other practical benefits—could make them suitable for use as adjunctive or supportive treatment in COVID-19 patients [190].

The use of adjunctive and prophylactic dietary therapies has also been promoted in the management of patients with COVID-19 [182]. As discussed previously, the gut microbiome is able to impact respiratory function via the gut–lung axis and diet can have a substantial impact on the composition of the gut microbiome [191]. Additionally, the intake of certain nutrients, such as vitamin C and zinc, has been associated with an anti-inflammatory effect, which could potentially be useful in managing patients with COVID-19 [180,192]. This has also led some to hypothesise that geographical differences in diet could be responsible for the variations in COVID-19 death rates between countries [193].

We need to reiterate that, however, it is difficult to judge how FMT, probiotics and dietary therapies should be used clinically in patients with SARS-CoV-2 infection, as the data regarding the efficacy of these treatments in this patient group is currently still very limited. More clinical trials are necessary before any concrete recommendations can be made on their use as adjunctive treatments for COVID-19.

### 6.6. Open Areas of Research

While there is still much about the impact of SARS-CoV-2 on the gut that is unknown, there is a great deal of research being conducted in this area, and Table 4 summarises some key research questions that still remain unanswered. One key area for future research will be establishing whether SARS-CoV-2 has a faecal–oral route of transmission and how this may impact the overall transmission rates. Additionally, further evidence is needed regarding whether microbial dysbiosis can predispose individuals to more severe COVID-19 disease progression. Additionally, it would be beneficial to measure the effects that potential COVID-19 treatments have on GI symptoms, as well as respiratory symptoms. Conversely, it is currently not clear which COVID-19 patients may benefit the most from gut-targeting therapies, and further research is required not only to confirm the efficacy of various therapies but also to satisfy the need to stratify which groups of patients would benefit the most.

Overall, it is likely that an understanding of the link between SARS-CoV-2 and the gut could be crucial to develop a more holistic understanding and treatment of COVID-19.

## 7. Conclusions

A functioning gut barrier plays an integral role in maintaining gut homeostasis and damage to this barrier has been associated with pathology and disease. Multiple therapies, that aim to improve intestinal permeability, have been explored. Many of these treatments are thought to have mechanisms of action that involve tight junction modulation. The association between the gut and COVID-19 highlights the importance of continuing research into therapies for gut pathology.

## Figures and Tables

**Table 1:** Examples of recent randomised-controlled trials that study the effect of non-dietary therapies on the intestinal barrier.

Reference	Therapy Studied	Organism(s) Studied	Results of Relevant Outcomes	Effect on Intestinal Permeability
Craven L et al., *Am. J. Gastroenterol*., 2020 [108]	FMT	n/a	Significant decrease in lactulose–mannitol ratio in treatment group	Decreased
Macnaughtan J et al., *Nutrients*, 2020 [119]	Probiotic	*Lactobacillus casei* Shirota	No significant difference between serum endotoxin concentrations of treatment and control group	No effect
Pugh JN et al., *Eur. J. Appl. Physiol.*, 2019 [121]	Probiotic	*Lactobacillus acidophilus, Bifidobacterium bifidum, Bifidobacterium animalis*	No significant difference between lactulose–rhamnose ratio of treatment and control group	No effect
Mokkala K et al., *Benef. Microbes*, 2018 [122]	Probiotic	*Bifidobacterium animalis, Lactobacillus rhamnosus*	No significant change in serum zonulin concentration in treatment group	No effect
Krumbeck JA et al., *Microbiome*, 2018 [128]	Probiotic	*Bifidobacterium*	Significant decrease in sucralose–lactulose ratio in some treatment groups	Decreased
Mujagic Z et al., *Sci. Rep.* 2017 [124]	Probiotic	*Lactobacillus plantarum*	No significant change in lactulose–rhamnose ratio in treatment	No effect
De Roos NM et al., *Eur. J. Clin. Nutr*., 2017 [120]	Probiotic	Multispecies probiotics	No significant change in lactulose–mannitol ratio, faecal zonulin concentration or serum zonulin concentration in treatment group	No effect
Liu ZH et al., *Am. J. Clin. Nutr.*, 2013 [116]	Probiotic	*Lactobacillus plantarum, Lactobacillus acidophilus, Bifidobacterium longum*	Significant reduction in serum zonulin concentration in treatment group	Decreased
Liu Z et al., *Aliment. Pharmacol. Ther*., 2011 [118]	Probiotic	*Lactobacillus plantarum, Lactobacillus acidophilus, Bifidobacterium longum*	Significant decrease in lactulose–mannitol ratio and bacterial translocation in treatment group	Decreased
Ramos CI et al., *Nephrol. Dial. Transplant*., 2019 [126]	Prebiotic	n/a	No significant difference between serum zonulin concentration of treatment and control group	No effect
Ho J et al., J. *Clin. Endocrinol. Metab*., 2019 [127]	Prebiotic	n/a	No significant difference between lactulose– mannitol ratio of treatment and control group	No effect
Krumbeck JA et al., *Microbiome*, 2018 [128]	Prebiotic	n/a	Significant decrease in sucralose–lactulose ratio in some treatment groups	Decreased

**Table 2:** Examples of randomised-controlled trials that study the effect of dietary therapies on the intestinal barrier that have been published in the last ten years.

Reference	Therapy Studied	Results of Relevant Outcomes	Effect on Intestinal Permeability
Wilms E et al., *Nutrients*, 2019 [129]	Dietary fiber	No significant change in lactulose-mannitol ratio in treatment group	No effect
Krawczyk M et al., *Nutrients*, 2018 [130]	Dietary fiber	Significant reduction in serum zonulin concentration in treatment group	Decreased
Zhou QQ et al., *Gut*, 2019 [131]	Glutamine	Significant decrease in lactulose-mannitol ratio in treatment group	Decreased
Benjamin J et al., *Dig. Dis. Sci*., 2012 [132]	Glutamine	Significant reduction in lactulose-mannitol ratio in treatment group, but a significant reduction was also seen in control group	Decreased
Lamprecht M et al., *J. Int. Soc. Sports Nutr.*, 2015 [133]	Zeolite supplements	Significant decrease in stool zonulin concentrations in treatment group	Decreased

**Table 3:** Examples of studies that investigate the link between SARS-CoV-2 and the gastrointestinal symptoms observed in COVID-19 patients (including alterations in the microbiome).

Reference	Study Topic	Relevant Conclusions of Study
Lamers MM et al., *Science*, 2020 [142]	SARS-CoV-2 infecting enterocytes	Found that SARS-CoV-2 can replicate in enterocytes. One way in which this was demonstrated was by using electron-microscopy to generate images of human small intestinal organoids that had been infected with SARS-CoV-2.
Lee JJ et al., *Genes* 2020 [145]	SARS-CoV-2 infecting enterocytes	Used colon samples of seven patients to conclude that TMPRSS2 and ACE2 are highly expressed in the lower GI tract.
Burgueno JF et al., *Inflamm. Bowel Dis.,* 2020 [146]	SARS-CoV-2 infecting enterocytes	ACE2 and TMPRSS2 are expressed in the intestinal epithelail cells of animal models.
Zang R et al., *Sci. Immunol.,* 2020 [168]	SARS-CoV-2 infecting enterocytes	Found that TMPRSS2 and TMPRSS4 facilitate the entry of SARS-CoV-2 into cells. Found that SARS-CoV-2 became inactivated by intestinal fluid. Stool samples did not show the presence of infectious SARS-CoV-2.
Cholankeril G et al., *Gastroenterology*, 2020 [150]	GI symptoms	Prevalence of gastrointestinal symptoms in patients with SARS-CoV-2 infection was 31.9% and 89.2% of these patients described their gastrointestinal symptoms as mild. It was found that AST levels were in correlation with disease activity.
Zheng T et al., *J. Med. Virol.*, 2020 [151]	GI symptoms	Rate of clinical decline was greater in patient with SARS-CoV-2 infection who had gastrointestinal symptoms in comparison to those who did not have gastrointestinal symptoms
Zhou Z et al., *Gastroenterology*, 2020 [153]	GI symptoms	Prevalence of gastrointestinal symptoms in patients with SARS-CoV-2 infection who had developed pneumonia was 26%. However, the presence of GI symptoms was not associated with clinical and treatment outcomes.
Redd WD et al., *Gastroenterology*, 2020 [154]	GI symptoms	Gastrointestinal symptoms were the main presenting complaint in 20.3% of patients with SARS-CoV-2 infection and 61.3% of those in the study reported experiencing a minimum of one GI symptom.
Ferm S et al., *Clin. Gastroenterol. Hepatol.*, 2020 [155]	GI Symptoms	Prevalence of gastrointestinal symptoms in patients with SARS-CoV-2 infection was 25%. It was found that higher levels of AST were associated with poorer health outcomes.
Nobel YR et al., *Gastroenterology*, 2020 [156]	GI symptoms	Prevalence of gastrointestinal symptoms in patients with SARS-CoV-2 infection was 35%. It was found that patients who presented with GI symptoms were 70% more likely to test positive for SARS-CoV-2.
Chen Y et al., *J. Med. Virol.*, 2020 [163]	GI symptoms	Prevalence of gastrointestinal symptoms in patients with SARS-CoV-2 infection was 19.05%. SARS-CoV-2 RNA was present in stool samples, but did not corrolate with presence of GI symptoms or disease severity.
Lin L et al., *Gut*, 2020 [166]	GI symptoms	Prevalence of gastrointestinal symptoms in patients with SARS-CoV-2 infection was 61.1%. Stool samples for hospitalisated patients were analysed and 47.7% tested positive for the presence of SARS-CoV-2.
Gu S et al., *Clin. Infect. Dis.*, 2020 [170]	Microbiome	The gut microbiome of COVID-19 patients is different to the gut microbiome of patients with H1N1 infection and healthy controls. COVID-19 patients had lower bacterial diversity than healthy controls and H1N1 patients had lower bacterial diversity than COVID-19 patients.
Yang T et al., *Hypertension*, 2020 [174]	Microbiome	Gut microbiota plays a role in the regulation of ACE-2 expression in the colon. This was shown through conducting gene sequencing of fecal samples collected from germ-free rats and conventionalized germ-free rats.
Zhang H et al., *Clin. Infect. Dis.*, 2020 [176]	Microbiome	Compared to those with non-COVID-19 pneumonias, COVID-19 patients appeared to have a more disrupted airway microbiome with frequent potential concurrent infections.
Effenberger M et al., *Gut*, 2020 [177]	Microbiome	Patients with COVID-19 are more likely to have a disrupted airway microbiome than pneumonia patients without COIVD-19.

**Table 4:** Some of the areas where further research is required to answer key questions to improve understanding about SARS-CoV-2 and the gut.

Area Requiring Further Research	Questions that Need To Be Answered
Faecal–oral transmission	Does SARS-CoV-2 have a faecal–oral route of transmission? How does this impact overall transmission rates?
Microbiome and immunity	Can microbial dysbiosis predispose individuals to more severe COVID-19 disease progression? How does local immune modulation by SARS-CoV-2 in the intestine affect systemic inflammatory responses, and how does this relate to COVID-19 severity and outcome?
Treatments and GI symptoms	What effect do treatments aimed at the respiratory symptoms of COVID-19 have on GI symptoms? Are therapies specifically targeting GI symptoms in COVID-19, such as FMT and probiotics, effective? Which groups of patients would benefit the most from receiving treatments that specifically target GI symptoms in COVID-19?
